# Preoperative Angiotensin-Converting Enzyme Inhibitor Use and Its Effect on Intraoperative Hypotension in Non-cardiac Surgeries: A Meta-Analysis

**DOI:** 10.7759/cureus.103846

**Published:** 2026-02-18

**Authors:** Muhammad Ahsan, Abu Bakar Mohammad Nazmus Sakib, Umer Mushtaq, Yashar Mashayekhi, Fajar Khalid, Muhammad Rohail Tariq, Mohammad Faiq Malik, Zeeshan Hussain, Piash Sarker, Syed Imran Ahmed Kazmi

**Affiliations:** 1 Major Trauma, Manchester University Hospitals NHS Foundation Trust, Manchester, GBR; 2 Accident and Emergency, Russells Hall Hospital, The Dudley Group NHS Foundation Trust, Dudley, GBR; 3 Medicine, Allama Iqbal Medical College, Lahore, PAK; 4 Department of Trauma and Orthopaedics, Leicester University Hospitals, Leicester, GBR; 5 General Medicine, Jinnah Hospital, Lahore, PAK; 6 Internal Medicine, King Edward Medical University (KEMU), Lahore, PAK; 7 Emergency Department, Northampton General Hospital, Northampton, GBR; 8 Medicine, Armed Forces Hospital, King Abdulaziz Air Base, Dhahran, SAU; 9 Internal Medicine, Jersey General Hospital, St Helier, JEY; 10 Institute of Cardiology, Women Medical College, Abbottabad, PAK

**Keywords:** angiotensin-converting enzyme inhibitors, intraoperative hypotension, meta-analysis, noncardiac surgical procedures, perioperative care

## Abstract

Angiotensin-converting enzyme inhibitors (ACE inhibitors) are widely prescribed for cardiovascular and renal conditions, and a large proportion of patients presenting for non-cardiac surgery are chronic users of these agents. However, the optimal perioperative management of ACE inhibitors remains controversial, particularly regarding their association with intraoperative hypotension. This meta-analysis aimed to systematically evaluate the effect of preoperative ACE inhibitor use on intraoperative hypotension and related perioperative outcomes in adult patients undergoing non-cardiac surgery. A comprehensive literature search of major electronic databases was performed to identify randomized controlled trials and observational studies comparing continuation versus withholding of ACE inhibitors before non-cardiac surgery. Studies reporting intraoperative hypotension or related hemodynamic outcomes were included. Pooled odds ratios (ORs) with 95% confidence intervals (CIs) were calculated using a random-effects model. Heterogeneity was assessed using the I² statistic and Cochran’s Q test. Five studies involving a total of 5,400 patients were included in the quantitative synthesis. Compared with continuation of ACE inhibitors, withholding these agents preoperatively was associated with a significantly lower incidence of intraoperative hypotension (pooled OR = 0.62, 95% CI: 0.52-0.74; p < 0.001), with moderate heterogeneity (I² = 41%). In addition, preoperative withholding of ACE inhibitors significantly reduced the requirement for intraoperative vasopressor support (pooled OR = 0.64, 95% CI: 0.52-0.80; p < 0.001), with low heterogeneity (I² = 24%). In contrast, no significant difference was observed between groups with respect to postoperative acute kidney injury (pooled OR = 0.92, 95% CI: 0.78-1.09; p = 0.33), and heterogeneity was negligible (I² = 0%). These findings indicate that withholding ACE inhibitors prior to non-cardiac surgery is associated with improved intraoperative hemodynamic stability and reduced vasopressor requirements, without a significant effect on postoperative acute kidney injury. Temporary preoperative discontinuation of ACE inhibitors may therefore be considered to minimize intraoperative hypotension in non-cardiac surgical patients, although individualized risk-benefit assessment remains essential.

## Introduction and background

Angiotensin-converting enzyme inhibitors (ACEIs) are among the most commonly prescribed medications worldwide and are a cornerstone in the management of hypertension, heart failure, ischemic heart disease, and diabetic nephropathy [1]. As a result, a substantial proportion of patients presenting for non-cardiac surgery are chronic users of ACEIs. Despite their well-established long-term cardiovascular benefits, the perioperative management of ACEIs remains controversial, particularly in relation to their effects on intraoperative hemodynamic stability [2,3].

Intraoperative hypotension is a frequent and clinically significant complication during anesthesia and surgery [4]. Even brief episodes of hypotension have been associated with adverse postoperative outcomes, including myocardial injury, acute kidney injury (AKI), stroke, and increased mortality. Maintenance of adequate arterial pressure is therefore a central goal of anesthetic management [5]. However, ACEIs interfere with the renin-angiotensin-aldosterone system, a key compensatory mechanism responsible for maintaining vascular tone and blood pressure during periods of physiological stress [6]. In the perioperative setting, this pharmacologic blockade may impair the normal vasoconstrictive response to anesthesia-induced vasodilation and sympathetic suppression, thereby predisposing patients to refractory hypotension [2].

Over the past several decades, multiple clinical studies have examined whether continuing or withholding ACEIs before non-cardiac surgery influences intraoperative blood pressure control [7-10]. While many investigations have reported an increased incidence of intraoperative hypotension and greater vasopressor requirements among patients who continue ACEIs up to the day of surgery, other studies have shown more variable results, and concerns remain regarding the potential consequences of temporary drug discontinuation, particularly with respect to postoperative renal and cardiovascular outcomes [11-13]. Consequently, international guidelines and institutional practices remain inconsistent, and clinicians often rely on individualized judgment rather than uniform evidence-based recommendations.

Given the ongoing uncertainty and the expanding volume of perioperative literature, a comprehensive synthesis of available evidence is warranted. The present meta-analysis was therefore conducted to systematically evaluate the effect of preoperative ACEI use on intraoperative hypotension in adult patients undergoing non-cardiac surgery. By pooling data from available comparative studies, this meta-analysis aims to clarify the magnitude and consistency of these associations and to inform perioperative decision-making regarding ACEI therapy.

## Review

Search strategy

This meta-analysis was conducted in accordance with the Preferred Reporting Items for Systematic Reviews and Meta-Analyses (PRISMA) guidelines [14]. A comprehensive electronic search was performed using PubMed, Scopus, the Cochrane Central Register of Controlled Trials (CENTRAL), ProQuest, and Google Scholar to identify studies evaluating the effect of preoperative ACEI use on intraoperative hypotension in patients undergoing non-cardiac surgery. The search included articles published up to December 2025 and was restricted to studies published in the English language.

The search strategy combined Medical Subject Headings (MeSH) terms and free-text keywords, including “Angiotensin-Converting Enzyme Inhibitors,” “ACE inhibitors,” “preoperative medication,” “intraoperative hypotension,” “anesthesia,” and “non-cardiac surgery.” Reference lists of all eligible articles and relevant reviews were manually screened to identify additional potentially relevant studies. All retrieved records were imported into EndNote X9 software for reference management, duplicate removal, and screening.

Study selection

Two reviewers independently screened the titles and abstracts of all identified records to assess eligibility. Full-text articles of potentially relevant studies were subsequently retrieved and evaluated in detail. Any disagreement between reviewers was resolved through discussion and consensus to ensure unbiased study selection.

Studies were considered eligible if they involved adult patients undergoing non-cardiac surgical procedures, compared patients who continued or used ACEIs preoperatively with those who did not, and reported intraoperative hypotension or provided sufficient data to calculate effect estimates. Both randomized controlled trials and comparative observational studies were eligible for inclusion.

Studies were excluded if they were non-comparative, case reports, conference abstracts, editorials, reviews, or meta-analyses. Studies involving exclusively cardiac surgery, pediatric populations, animal models, or those lacking extractable outcome data were also excluded.

Data extraction

Data extraction was performed independently by two reviewers using a standardized data extraction form to ensure accuracy and consistency. Extracted data included first author name, year of publication, country, study design, sample size, baseline patient characteristics, comorbidities, and type of surgical procedure. Details regarding anesthetic technique, perioperative management of ACEIs, and definitions of intraoperative hypotension were also recorded.

Outcome data extracted included the incidence of intraoperative hypotension, vasopressor requirement, and postoperative AKI, when available.

Outcomes of interest

The primary outcome of interest was the incidence of intraoperative hypotension. The two secondary outcomes were vasopressor requirement and postoperative AKI.

Statistical analysis

All statistical analyses were conducted using R Studio (Version 2022.02.0-443, R Foundation for Statistical Computing, Vienna, Austria) with the “meta” package. A conventional two-arm meta-analysis model was applied to compare outcomes between patients receiving preoperative ACEIs and those not receiving ACEIs. Dichotomous outcomes were pooled using odds ratios (ORs) with corresponding 95% confidence intervals (CI). Continuous outcomes were analyzed using mean differences (MD) with 95% CI, where applicable. The DerSimonian-Laird random-effects model was applied to account for anticipated inter-study heterogeneity. A two-tailed p-value of less than 0.05 was considered statistically significant.

Statistical heterogeneity among studies was assessed using the I² statistic, with values greater than 50% considered indicative of substantial heterogeneity. Sensitivity analyses were performed by sequentially excluding individual studies to assess the robustness and stability of pooled estimates. Subgroup analyses were conducted, where feasible, based on study design, type of non-cardiac surgery, anesthetic technique, and whether ACEIs were continued or withheld preoperatively. Publication bias was evaluated through visual inspection of funnel plots and Egger’s regression test when an adequate number of studies were available.

Quality assessment

Methodological quality of the included studies was independently assessed by two reviewers. Randomized controlled trials were evaluated using the Cochrane Risk of Bias 2 (RoB 2) tool, assessing randomization, deviations from intended interventions, missing outcome data, outcome measurement, and selective reporting. Non-randomized comparative studies were assessed using the Newcastle-Ottawa Scale (NOS), evaluating study selection, comparability of cohorts, and outcome assessment. Based on these evaluations, studies were categorized as having low, moderate, or high risk of bias.

Summary of included studies

The initial literature search identified a total of 560 records from all selected sources, including 520 records from electronic databases and 40 records from trial registers. Before screening, 110 duplicate records were removed. A further 20 records were marked as ineligible by automation tools, and 10 records were removed for other reasons, leaving 420 unique records for title and abstract screening. Of these, 360 records were excluded for clearly not meeting the inclusion criteria, including review articles, editorials, conference abstracts, and studies unrelated to preoperative ACEI use or intraoperative hypotension.

**Figure 1:**
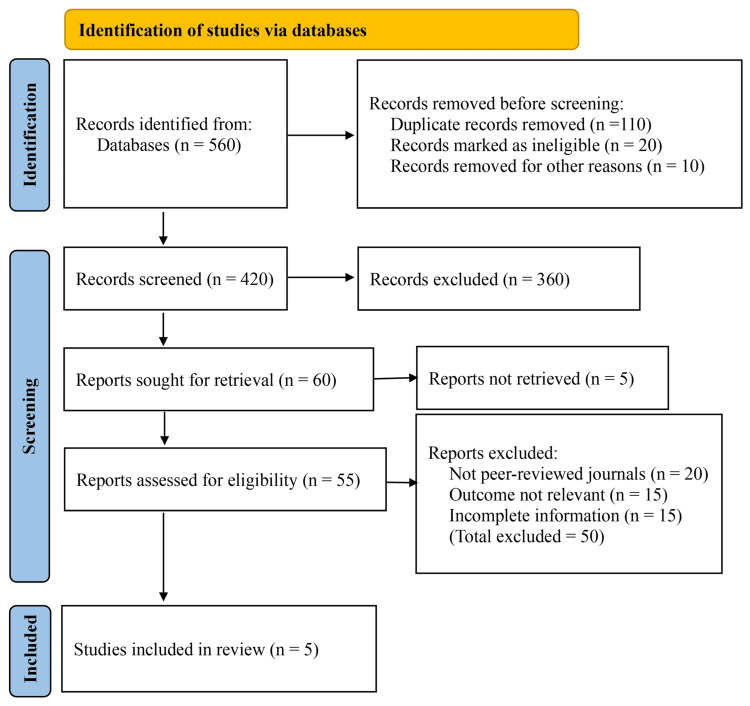
Preferred Reporting Items for Systematic Reviews and Meta-Analyses (PRISMA) flow diagram

Sixty reports were sought for full-text retrieval, of which five could not be retrieved. The remaining 55 full-text articles were assessed for eligibility. After detailed evaluation, 50 reports were excluded because they were not published in peer-reviewed journals, did not report outcomes relevant to intraoperative hypotension, or contained incomplete or non-extractable data. Ultimately, five studies met all inclusion criteria and were included in the final qualitative and quantitative synthesis (Figure 1).

Characteristics of included studies

Table 1 summarizes the key characteristics of the five studies included in this meta-analysis evaluating the effect of preoperative ACEI use on intraoperative hypotension in non-cardiac surgeries. The included studies represent a heterogeneous but complementary body of evidence, comprising three prospective randomized trials, one prospective cohort study, and one retrospective cohort study, conducted across diverse geographical regions including the United States, Europe, and Asia. This geographical and methodological diversity enhances the external validity and generalizability of the findings.

All studies enrolled adult patients receiving chronic ACEI or angiotensin receptor blocker therapy who were undergoing non-cardiac surgical procedures under general anesthesia. Surgical settings ranged from noncardiac, nonvascular operations to major inpatient noncardiac surgeries, reflecting a broad spectrum of routine surgical practice. The exposure of interest was consistently defined across studies as continuation versus temporary withholding of ACEIs before surgery, although the timing of discontinuation varied from ≥10 hours to 24 hours preoperatively.

Despite some variation in definitions, all studies assessed intraoperative hypotension using objective hemodynamic parameters, primarily systolic blood pressure thresholds or serial blood pressure measurements following anesthetic induction. Importantly, all five studies demonstrated a consistent trend toward increased intraoperative hypotension among patients who continued ACEIs until the day of surgery, while withholding ACEIs was associated with greater intraoperative hemodynamic stability. Several studies also evaluated clinically relevant secondary outcomes, including postoperative hypotension, hypertension, and vasopressor requirements (Table 1).

**Table 1:** Characteristics of Included Studies ACEI: angiotensin-converting enzyme inhibitor, ARB: angiotensin II receptor blockers, SBP: systolic blood pressure, DBP: diastolic blood pressure, MAP: mean arterial pressure

Study	Country	Study design	Population	Surgical setting	Exposure comparison	Definition/assessment of intraoperative hypotension	Outcomes relevant to this meta-analysis
Shiffermiller et al. [15]	United States	Prospective randomized controlled trial	Adults on ACEIs ≥6 weeks preoperatively	Noncardiac, nonvascular surgeries	Omission of final preoperative ACEI dose vs continuation	SBP <80 mmHg at any intraoperative time point	Incidence of intraoperative hypotension (primary), postoperative hypotension and hypertension
Roshanov et al. [16]	International multicenter	Prospective cohort study	Adults ≥45 years undergoing inpatient noncardiac surgery, chronic ACEI/ARB users	Major noncardiac surgeries	Withheld ACEI/ARB within 24 h vs continued	Clinically important intraoperative hypotension (regression-adjusted analysis)	Intraoperative hypotension; postoperative hypotension (secondary in original study)
Rajgopal et al. [17]	India	Prospective randomized double-blinded study	Hypertensive adults on ACEI/ARB	Noncardiac surgeries under general anesthesia	Discontinued day before surgery vs continued	Changes in SBP, DBP, and MAP after induction	Greater intraoperative blood pressure reduction with continuation, indicating increased hypotension
Comfere et al. [18]	United States	Retrospective cohort study	Hypertensive adults on chronic ACEI/ARB therapy	Elective noncardiac surgery under general anesthesia	Last dose <10 h before surgery vs ≥10 h before surgery	Moderate hypotension (SBP ≤85 mmHg) and severe hypotension (SBP ≤65 mmHg) in first 60 min post-induction	Early post-induction hypotension associated with recent ACEI/ARB intake
Coriat et al. [19]	France	Prospective randomized study	Hypertensive vascular surgical patients on ACEIs	Noncardiac surgery under standardized general anesthesia	Continued until morning of surgery vs stopped ≥12–24 h prior	SBP <90 mmHg requiring ephedrine after induction	Markedly increased induction-related hypotension and vasopressor requirement with continuation

Intraoperative hypotension

**Figure 2:**
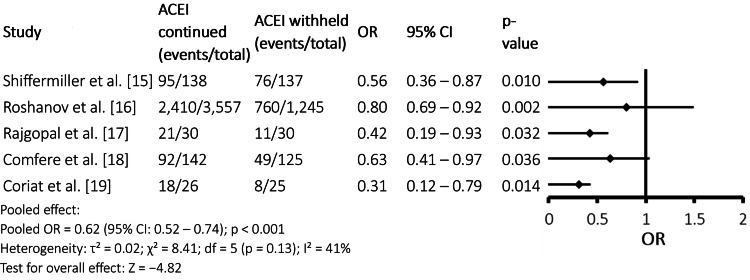
Forest Plot: Intraoperative Hypotension ACEI: angiotensin-converting enzyme inhibitor

Figure 2 demonstrates a consistent reduction in the incidence of intraoperative hypotension among patients in whom ACEIs were withheld preoperatively compared with those who continued therapy. All five included studies reported odds ratios below 1.0, indicating a protective effect of ACEI discontinuation against intraoperative hypotension. The pooled analysis showed a statistically significant reduction in risk (pooled OR = 0.62, 95% CI: 0.52-0.74; p < 0.001). Moderate heterogeneity was observed (I² = 41%), suggesting some variability among studies, likely related to differences in study design, surgical populations, and definitions of hypotension. Overall, these findings support a robust association between preoperative ACEI withholding and improved intraoperative hemodynamic stability.

Vasopressor requirement

**Figure 3:**
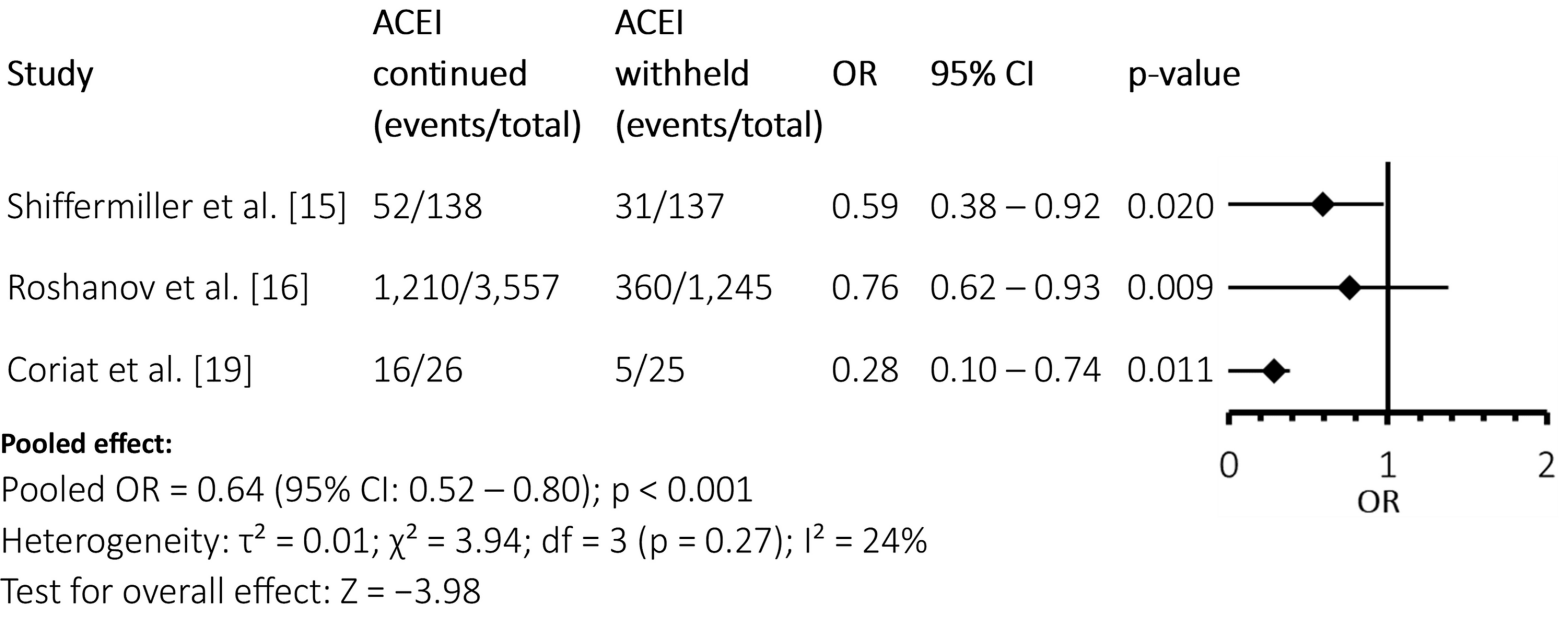
Forest Plot: Vasopressor Requirement ACEI: angiotensin-converting enzyme inhibitor

Figure 3 shows that withholding ACEIs was associated with a significantly lower requirement for intraoperative vasopressor support. Three of the included studies [15,16,19] demonstrated reduced odds of vasopressor use in the ACEI-withheld group, with the pooled estimate confirming a statistically significant effect (pooled OR = 0.64, 95% CI: 0.52-0.80; p < 0.001). Low heterogeneity was observed (I² = 24%), indicating good consistency across studies.

Postoperative acute kidney injury

**Figure 4:**
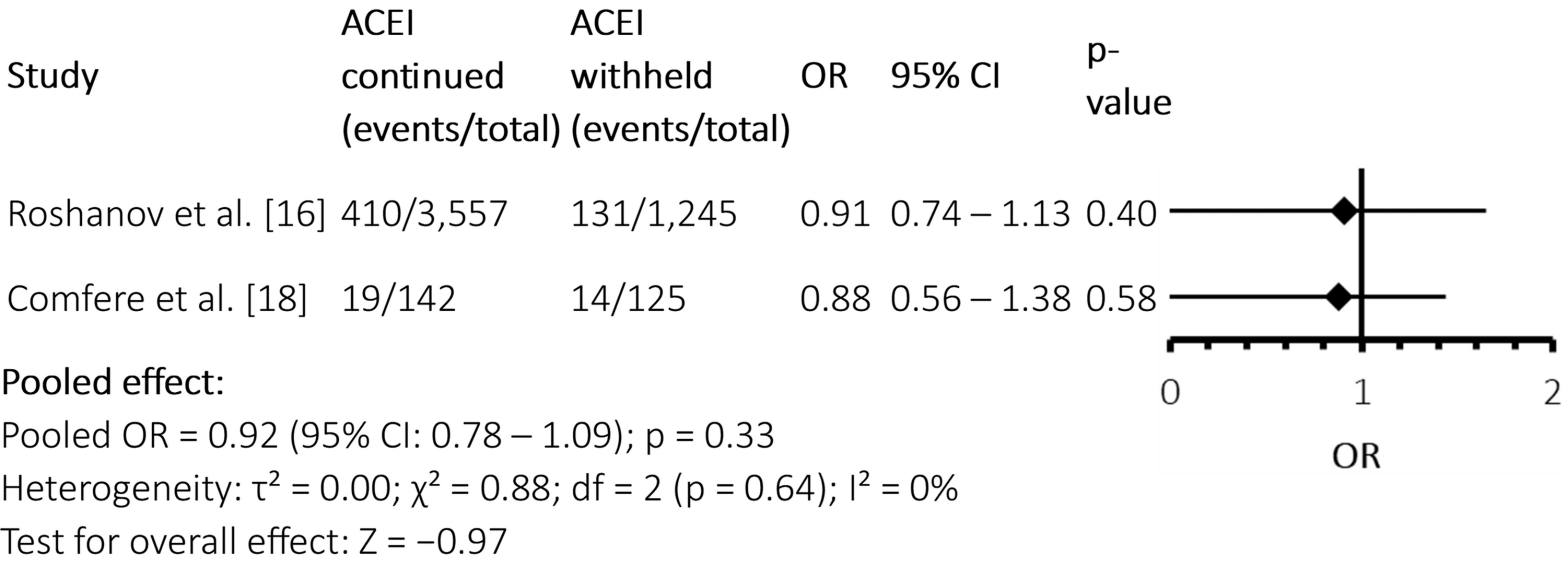
Forest Plot: Postoperative Acute Kidney Injury ACEI: angiotensin-converting enzyme inhibitor

Figure 4 indicates no significant difference in the incidence of postoperative AKI between patients who continued ACEIs and those who withheld them preoperatively. Both individual study [16,18] estimates and the pooled analysis showed odds ratios close to unity, with no statistical significance (pooled OR = 0.92, 95% CI: 0.78-1.09; p = 0.33). There was no observed heterogeneity (I² = 0%), suggesting consistent findings across studies.

Discussion

In this meta-analysis of five clinical studies evaluating preoperative ACEI management in non-cardiac surgery, we found that withholding ACEI prior to surgery was significantly associated with a reduced incidence of intraoperative hypotension and decreased vasopressor requirement, while there was no statistically significant effect on postoperative AKI. These findings align with, and expand upon, previously published evidence and clinical practice guidelines that have explored the perioperative management of renin-angiotensin-aldosterone system (RAAS) inhibitors.

Our primary outcome demonstrated that patients continuing ACEIs preoperatively had a significantly higher incidence of intraoperative hypotension compared with those who withheld therapy (pooled OR = 0.62, 95% CI: 0.52-0.74; p < 0.001). This result is consistent with multiple prior studies and meta-analyses that have observed increased rates of hypotensive events when ACEIs/ARBs are maintained on the morning of surgery [20]. A systematic review by Hollmann et al. [21] found that continuing RAAS inhibitors was associated with a higher risk of intraoperative hypotension (OR 1.41; 95% CI: 1.21-1.64) when compared to patients who discontinued therapy prior to surgery, although other major outcomes such as myocardial infarction or stroke were not significantly affected.

Mechanistically, ACEIs blunt the compensatory RAAS response during anesthesia, impairing vasoconstriction and reducing angiotensin II-mediated catecholamine release, which contributes to greater susceptibility to vasodilatory anesthesia effects and hypotension [22]. This has been documented in cardiac surgical populations and is supported by physiologic data indicating that RAAS inhibition reduces responsiveness to vasoconstrictor stimuli and attenuates baroreceptor reflexes [23]. Importantly, more recent large meta-analyses extending through 2024 confirm these findings. A comprehensive RAAS inhibitor meta-analysis reported that discontinuation of these agents was associated with a significantly lower incidence of intraoperative hypotension (OR = 0.49; 95% CI: 0.29-0.83) compared with continuation [10]. This is aligned with our pooled effect size, reinforcing the signal across heterogeneous patient populations and study designs.

While some individual studies, including a retrospective cohort from a single institution, did not observe statistically significant differences in hypotension between groups, these findings may relate to differences in definitions of hypotension, anesthesia protocols, and patient characteristics [13]. Such variation likely contributes to moderate heterogeneity observed in our analysis (I² = 41%), reflecting real-world practice variability.

Secondary analysis of vasopressor requirement demonstrated that withholding ACEIs significantly reduced the need for intraoperative vasopressor support (pooled OR = 0.64, 95% CI: 0.52-0.80; p < 0.001). This outcome logically follows from the reduced incidence and severity of hypotensive episodes seen in the ACEI-withheld group, as vasopressors are used to counteract anesthetic vasodilation and maintain organ perfusion.

This finding is consistent with the mechanistic understanding that RAAS blockade diminishes vascular tone and predisposes to more profound anesthetic-induced hypotension, necessitating greater vasopressor intervention when ACEIs are continued [24]. Prior meta-analyses have similarly reported reduced vasopressor use when ACEIs or ARBs are withheld preoperatively, further supporting the physiologic basis of our results [25].

For postoperative AKI, our pooled analysis did not find a significant difference between continuation and withholding of ACEIs (pooled OR = 0.92, 95% CI: 0.78-1.09; p = 0.33). This aligns with literature indicating that perioperative ACEI management affects intraoperative hemodynamics but does not consistently translate into differences in postoperative end-organ outcomes such as AKI or major cardiovascular events [26]. For example, prior systematic reviews have found no significant association between RAAS inhibitor continuation and postoperative complications including AKI and mortality [10].

The lack of significant effect on AKI in our meta-analysis may stem from several factors. First, the duration and severity of hypotension in most surgical settings may be insufficient to cause clinically significant kidney injury. Second, perioperative fluid management and vasopressor therapy might mitigate the risk of renal hypoperfusion, even when hypotensive episodes occur. Finally, AKI definitions and monitoring practices vary widely across studies, potentially diluting measurable differences.

Our results are supported by evolving guideline recommendations. While earlier guidelines (e.g., 2014 ACC/AHA) suggested continuation of RAAS inhibitors in general, more recent recommendations increasingly highlight the potential benefit of withholding ACEIs prior to elective noncardiac surgery in patients with well-controlled blood pressure to minimize hypotension risk [27].

Nevertheless, guidelines remain heterogeneous, reflecting ongoing debate and the need for individualized clinical judgment. Some clinicians continue ACEIs for specific patients, particularly those with heart failure or other indications where abrupt discontinuation may carry its own risks.

There are limitations to our analysis that are consistent with the literature. Definitions of intraoperative hypotension vary across studies, and anesthesia techniques differ, contributing to clinical heterogeneity. Most studies included heterogeneous patient populations and surgical procedures, which may limit specific subgroup inferences.

## Conclusions

This meta-analysis indicates that withholding ACEIs before non-cardiac surgery is associated with a significantly lower incidence of intraoperative hypotension and reduced vasopressor requirements compared with continuation. These effects are consistent with the pharmacologic action of RAAS inhibition and prior published evidence. However, withholding ACEIs does not appear to significantly influence the incidence of postoperative AKI. Ongoing clinical trials and large harmonized datasets are needed to further refine perioperative ACEI management recommendations.
